# Clinician-Level Knowledge and Barriers to Hepatocellular Carcinoma Surveillance

**DOI:** 10.1001/jamanetworkopen.2024.11076

**Published:** 2024-05-14

**Authors:** Robert J. Wong, Patricia D. Jones, Bolin Niu, George Therapondos, Mae Thamer, Onkar Kshirsagar, Yi Zhang, Paulo Pinheiro, Beverly Kyalwazi, Ronnie Fass, Mandana Khalili, Amit G. Singal

**Affiliations:** 1 Division of Gastroenterology and Hepatology, Stanford University School of Medicine and Gastroenterology Section, Veterans Affairs Palo Alto Healthcare System, Palo Alto, California; 2 Division of Digestive Health and Liver Diseases, University of Miami School of Medicine and Jackson Memorial Health System, Miami, Florida; 3 Division of Gastroenterology and Hepatology, MetroHealth Hospital and Health System, Cleveland, Ohio; 4 Multiorgan Transplant Institute, Ochsner Health, New Orleans, Louisiana; 5 Medical Technology and Practice Patterns Institute, Bethesda, Maryland; 6 Division of Epidemiology and Population Health Sciences, University of Miami School of Medicine, Miami, Florida; 7 Department of Medicine, University of Texas Southwestern Medical Center, Dallas; 8 Division of Gastroenterology and Hepatology, University of California San Francisco and Zuckerberg San Francisco General Hospital, San Francisco; 9 Division of Digestive and Liver Diseases, University of Texas Southwestern Medical Center and Parkland Health, Dallas

## Abstract

**Importance:**

Surveillance for hepatocellular carcinoma (HCC) in patients with cirrhosis is underused. Identifying potentially modifiable factors to address barriers in HCC surveillance is critical to improve patient outcomes.

**Objective:**

To evaluate clinician-level factors contributing to underuse of HCC surveillance in patients with cirrhosis.

**Design, Setting, and Participants:**

This survey study included primary care clinicians (PCCs) and gastroenterology and hepatology clinicians at 5 safety-net health systems in the US. Clinicians were surveyed from March 15 to September 15, 2023, to assess knowledge, attitudes, beliefs, perceived barriers, and COVID-19–related disruptions in HCC surveillance in patients with cirrhosis. Data were analyzed from October to November 2023.

**Main Outcome and Measures:**

HCC surveillance knowledge was assessed with 6 questions querying the respondent’s ability to correctly identify appropriate use of HCC surveillance. Attitudes, perceived barriers, and beliefs regarding HCC surveillance and perceived impact of the COVID-19 pandemic–related disruptions with HCC surveillance were assessed with a series of statements using a 4-point Likert scale and compared PCCs and gastroenterology and hepatology clinicians.

**Results:**

Overall, 347 of 1362 clinicians responded to the survey (25.5% response rate), among whom 142 of 237 (59.9%) were PCCs, 48 of 237 (20.3%) gastroenterology and hepatology, 190 of 236 (80.5%) were doctors of medicine and doctors of osteopathic medicine, and 46 of 236 (19.5%) were advanced practice clinicians. On HCC knowledge assessment, 144 of 270 (53.3%) scored 5 or more of 6 questions correctly, 37 of 48 (77.1%) among gastroenterology and hepatology vs 65 of 142 (45.8%) among PCCs (*P* < .001). Those with higher HCC knowledge scores were less likely to report barriers to HCC surveillance. PCCs were more likely to report inadequate time to discuss HCC surveillance (37 of 139 [26.6%] vs 2 of 48 [4.2%]; *P* = .001), difficulty identifying patients with cirrhosis (82 of 141 [58.2%] vs 5 of 48 [10.4%]; *P* < .001), and were not up-to-date with HCC surveillance guidelines (87 of 139 [62.6%] vs 5 of 48 [10.4%]; *P* < .001) compared with gastroenterology and hepatology clinicians. While most acknowledged delays during the COVID-19 pandemic, 62 of 136 PCCs (45.6%) and 27 of 45 gastroenterology and hepatology clinicians (60.0%) reported that patients with cirrhosis could currently complete HCC surveillance without delays.

**Conclusions and Relevance:**

In this survey study, important gaps in knowledge and perceived barriers to HCC surveillance were identified. Effective delivery of HCC education to PCCs and health system–level interventions must be pursued in parallel to address the complex barriers affecting suboptimal HCC surveillance in patients with cirrhosis.

## Key Points

**Question:**

Which clinician-level factors contribute to underuse of hepatocellular carcinoma (HCC) surveillance in patients with cirrhosis?

**Findings:**

In this survey study of 347 primary care clinicians (PCCs) and gastroenterology and hepatology clinicians across 5 US safety-net health systems, gaps in HCC surveillance knowledge were identified, particularly among PCCs. Perceived barriers and challenges to HCC surveillance use were identified, including health system factors contributing to persisting delays in timely HCC surveillance despite 3 years after the onset of the COVID-19 pandemic.

**Meaning:**

These findings suggest that improved HCC education, particularly for PCCs, and health system–level interventions must be pursued in parallel to address the complex barriers contributing to suboptimal HCC surveillance.

## Teaser

This survey study evaluates knowledge of, attitudes toward, and barrier to hepatocellular cancer (HCC) surveillance among safety-net hospitals in the US.

## Introduction

Hepatocellular carcinoma (HCC) is a leading cause of morbidity and mortality globally.^1,2^ In the US, the American Cancer Society estimates 41 210 new cases of liver cancer will be diagnosed and 29 380 people will die from liver cancer in 2023.^3^ Progression of underlying chronic liver disease to cirrhosis is the main risk factor for development of HCC, and the incidence of HCC among individuals who have developed cirrhosis exceeds 1.0% per year, a threshold above which current guidelines recommend HCC surveillance.^2^

Effective HCC surveillance in patients with cirrhosis leads to an earlier tumor stage at the time of diagnosis, which translates into more options for potentially curative treatment and improved overall survival.^4,5,6,7^ However, the use of HCC surveillance among patients with cirrhosis remains suboptimal. A recent systematic review and meta-analysis^8^ that included 29 studies with a total of 118 799 patients with cirrhosis reported a pooled estimate for surveillance use of 24.0%. Similarly, a recent analysis^9^ in a cohort of more than 2000 patients with cirrhosis found the proportion of time covered by surveillance was 24.9%, with only 16% of patients having semiannual surveillance in the year before HCC diagnosis. Low HCC surveillance use in patients with cirrhosis along with data from the National Cancer Institute’s Surveillance Epidemiology and End Results database showing overall 5-year survival in patients with HCC less than 30% highlights an urgent need to improve HCC surveillance.

Underlying causes for low HCC surveillance use are complex and multifactorial, and likely reflect patient, clinician, and system-level factors.^10,11^ Existing studies^12,13,14,15,16,17^ evaluating clinician-level factors have identified gaps in clinician knowledge or familiarity with HCC surveillance guidelines as potential contributors to low rates of HCC surveillance. Studies^13,15,17^ focusing predominantly on primary care clinicians (PCCs, including family and internal medicine clinicians) have also identified misconceptions and perceived barriers and attitudes toward HCC surveillance as potential contributors to low rates of surveillance use in patients with cirrhosis. Safety-net health systems care for predominantly low socioeconomic status populations and ethnic minorities, who are disproportionately impacted by HCC and experience increased HCC mortality.^18,19,20^ HCC surveillance is lower in these groups, contributing to these worse outcomes.^17,21,22,23,24,25^ Hence studies aimed at better understanding contributors to HCC surveillance specifically among safety-net populations have immense potential to drive interventions to improve HCC surveillance and HCC outcomes in patients with cirrhosis. In particular, elucidating clinician-level factors contributing to underuse or delays in implementation of HCC surveillance is critical to identify potentially modifiable factors, opportunities for education, or other targeted inventions to improve HCC surveillance in patients with cirrhosis. We surveyed PCC and gastroenterology and hepatology clinicians across 5 safety-net health systems in the US to better understand clinician-level factors that may contribute to underuse of HCC surveillance in patients with cirrhosis.

## Methods

This survey study was approved by the institutional review boards (IRBs) of each respective institution and health system. We followed the Consensus-Based Checklist for Reporting of Survey Studies (CROSS). A waiver of documentation of informed consent was granted because this survey study was minimal risk and anonymous.

### Study Population

We conducted an anonymous, online web-based survey to a convenience sample of PCCs (internal medicine or family practice) and gastroenterology and hepatology clinicians across 5 safety-net health systems in California, Florida, Louisiana, Ohio, and Texas. We included clinicians who identified themselves as attending physicians, advanced practice clinicians, and residents or fellows.

### Survey Information

The survey was developed using a conceptual model based on Social Cognitive Theory,^26^ Theory of Reasoned Action,^27^ and Theory of Planned Behavior,^28^ which has been previously used and adapted to evaluate clinician practice patterns related to HCC surveillance in cirrhosis^13,15,16^ The questions included in the survey (eMethods in Supplement 1) aimed to assess knowledge, attitudes, beliefs, perceived barriers, and COVID-19–related disruptions in HCC surveillance in patients with cirrhosis. The survey was divided in 6 sections and included a combination of questions and clinical scenarios with multiple choice answers to assess recommended HCC surveillance strategies (eg, when to initiate surveillance, frequency of surveillance, modality to implement surveillance), and a series of statements assessing attitudes, perceptions, and beliefs regarding HCC surveillance using a 4-point Likert Scale. An additional set of questions assessed how the COVID-19 pandemic and transitions in care affected HCC surveillance. Demographic characteristics, such as gender and self-reported race and ethnicity, were also collected. Race and ethnicity data were collected because other studies have suggested that race and ethnicity concordance between patient and clinician may influence a patient's receptiveness of completing recommended testing. Categories were American Indian or Alaska Native, Asian, Black or African American, Native Hawaiian or other Pacific Islander, White, and other.

 The survey took approximately 15 minutes to complete. Invitations to participate in the survey were distributed via email between March 15 and September 15, 2023, with an embedded survey link to a REDCap-linked survey. After the initial email invitation, reminder emails were sent every 2 weeks for a total of 6 weeks.

### Statistical Analysis

Descriptive statistics of the survey respondents used frequency and proportions. Not all survey respondents completed all of the questions in the survey. The denominator used to calculate proportions for each question or category was based on the total number of respondents for each question and excluded those who did not answer the question. The denominator used to calculate the proportions for each question or category are indicated in the first column of each table. HCC surveillance knowledge was assessed with 6 questions that queried the respondent’s ability to correctly identify timing of guideline-concordant surveillance and the diagnostic modality with which to perform surveillance (eTable 2 in Supplement 1). One point was given for each correct answer, with a total of 6 possible points for complete HCC surveillance knowledge assessment. Based on the distribution of responses, comparisons of the proportion of respondents answering 5 to 6 questions correctly vs 0 to 4 questions correctly were stratified by clinician characteristics and analyzed with χ^2^ methods. Perceived barriers to HCC surveillance based on previous studies were assessed with 8 statements that evaluated factors, such as adequate time during visits to discuss surveillance, language barriers, lack of diagnostic radiology resources, concerns regarding patient out-of-pocket costs, and challenges arranging follow-up testing and/or treatment. The proportion of respondents endorsing 1 or more of these barriers were similarly stratified by patient characteristics and compared using χ^2^ methods. Next, we sought to understand whether there were major differences in perceived barriers to HCC surveillance between PCCs (internal medicine and family medicine clinicians) and gastroenterology and hepatology clinicians. Differences in perceived barriers to HCC surveillance as well as beliefs, attitudes, and perceptions toward HCC surveillance were stratified by PCC vs gastroenterology and hepatology clinicians. Similarly, the perceived impact of disruptions associated with the COVID-19 pandemic and HCC surveillance among patients with cirrhosis were compared between PCCs and gastroenterology and hepatology clinicians using χ^2^ methods. Statistical analyses were performed using SAS Studio 3.6 on SAS version 9.4 (SAS Institute). Statistical significance was met with a 2-tailed *P* < .05. Data were analyzed from October to November 2023.

## Results

### Clinician Characteristics

Of the 1362 clinicians who were invited to participate, 347 responded (25.5% response rate). Among the 237 clinicians who answered the question regarding clinician specialty, 142 (59.9%) identified as PCCs, 48 (20.3%) identified as gastroenterology or hepatology clinicians, 39 (16.5%) were residents or fellows, and 8 (3.4%) were others (eTable 1 in Supplement 1). Most clinicians (190 of 236 [80.5%]) were doctors (ie, doctors of medicine and doctors of osteopathic medicine), followed by 46 of 236 (19.5%) who identified as nurse practitioners or physician assistants. Most respondents were women (148 of 233 [63.5%]) and 50 of 232 (21.5%) identified as Asian; 18 of 232 (7.8%) as Black; and 147 of 242 (63.4%) as White. Additionally, 151 of 310 clinicians (48.1%) reported having 10 or more years of clinical experience, and 154 of 309 clinicians (49.8%) reported seeing an average of 50 or more patients per week.

### Clinician Knowledge of Surveillance

Among respondents who completed the HCC surveillance knowledge questions, 144 of 270 (53.3%) scored at least 5 of 6 correct (Table 1 and eTable 2 in Supplement 1). A higher proportion of gastroenterology and hepatology clinicians had higher surveillance knowledge compared with PCCs (37 of 28 [77.1%] vs 65 of 142 [45.8%]; *P* < .001), and clinicians with less than 10 years of clinical experience had higher knowledge vs those with more than 20 years of experience (84 of 161 [52.2%] vs 29 of 82 [35.4%]; *P* = .046). Clinicians who reported seeing 25 to 49 patients weekly had the greatest HCC surveillance knowledge, which was higher than clinicians who reported seeing 50 or more patients per week (59 of 97 [60.8%] vs 54 of 154 [35.1%]; *P* < .001). No significant differences in HCC surveillance knowledge score were observed by gender or clinician type or training.

**Table 1.** Differences in HCC Surveillance Knowledge by Clinician Characteristics

Characteristics	Respondents, No. (%)^a^		*P* value
	≤4 Correct^b^	5 or 6 Correct^b^	
Total (N = 270)	126 (46.6)	144 (53.3)	NA
Clinician specialty (n = 190)			
Internal medicine or family medicine	77 (54.2)	65 (45.8)	<.001
Gastroenterology and hepatology	11 (22.9)	37 (77.1)	
Gender (n = 233)			
Men	34 (40.0)	51 (60.0)	.38
Women	68 (45.9)	80 (54.1)	
Clinician type/training (n = 236)			
MD or DO in training	14 (33.3)	28 (66.7)	.14
MD or DO	65 (43.9)	83 (56.1)	
NP or PA	25 (54.3)	21 (45.7)	
Clinician experience, y (n = 310)			
<10	77 (47.8)	84 (52.2)	.046
10-20	36 (53.7)	31 (46.3)	
>20	53 (64.6)	29 (35.4)	
No. patients in a typical week (n = 309)			
<25	28 (48.3)	30 (51.7)	<.001
25-49	38 (39.2)	59 (60.8)	
50	100 (64.9)	54 (35.1)	

Abbreviations: DO, doctor of osteopathic medicine; HCC, hepatocellular carcinoma; MD, medical doctor; NA, not applicable; NP, nurse practitioner; PA, physician assistant.

^a^ Not all respondents answered all questions. Calculation of proportions were based on the total number of clinicians who answered each question. The number in parentheses in the first column indicates the total number of respondents for each category.

^b^ The 6 questions on the HCC surveillance are available in eTable 2 in Supplement 1.

### Clinician Barriers to Surveillance

PCCs were more likely to report barriers to HCC surveillance compared with gastroenterology or hepatology clinicians (64 of 141 [46.4%] vs 11 of 43 [25.6%]; *P* = .02) (Table 2). In addition, clinicians with lower HCC surveillance knowledge were more likely to report barriers to surveillance (66 of 112 [58.9%] vs 35 of 131 [26.7%]; *P* < .001).

**Table 2.** Differences in Perceived Barriers to HCC Surveillance by Clinician Characteristics

Characteristics	Respondents, No. (%)^a^		*P* value
	No barriers reported^b^	Barriers reported^b^	
Total (n = 243)	142 (58.4)	101 (41.6)	NA
Clinician specialty (n = 181)			
Internal medicine or family medicine	74 (53.6)	64 (46.4)	.02
Gastroenterology and hepatology	32 (74.4)	11 (25.6)	
Gender (n = 222)			
Men	44 (54.3)	37 (45.7)	.28
Women	87 (61.7)	54 (38.3)	
Clinician type or training (n = 225)			
MD or DO in training	28 (70.0)	12 (30.0)	.24
MD or DO	78 (55.3)	63 (44.7)	
NP or PA	27 (61.4)	17 (38.6)	
Clinician experience, y (n = 243)			
<10^c^	83 (64.8)	45 (35.2)	.10
10-20	27 (50.0)	27 (50.0)	
>20	32 (52.5)	29 (47.5)	
How many patients in a typical week (n = 242)			
<25	28 (60.9)	18 (39.1)	.28
25-49	53 (64.6)	29 (35.4)	
≥50	61 (53.5)	53 (46.5)	
HCC Knowledge Score (n = 243)^d^			
≤4	46 (41.1)	66 (58.9)	<.001
5-6	96 (73.3)	35 (26.7)	

Abbreviations: DO, doctor of osteopathic medicine; HCC, hepatocellular carcinoma; MD, medical doctor; NA, not applicable; NP, nurse practitioner; PA, physician assistant.

^a^ Not all respondents answered all questions. Calculation of proportions were based on the total number of clinicians who answered each question. The number in parentheses in the first column indicates the total number of respondents for each category.

^b^ No barriers reported indicates those who answered no barriers to all questions in Table 3. Barriers reported indicated those who answered yes to barriers for any question in Table 3.

^c^ Including those in training.

^d^ Out of 6 correct.

Overall, the most commonly reported barriers among all respondents included not having adequate time to discuss HCC surveillance (39 of 187 [20.9%]), concerns about patients’ out-of-pocket costs (27 of 187 [14.4%]), and difficulty arranging follow-up diagnostic testing for patients with a positive HCC screening test (17 of 186 [9.1%]). Table 3 compares specific barriers reported by PCCs and gastroenterology and hepatology clinicians. PCCs, compared with gastroenterology and hepatology clinicians, were more likely to report barriers in having adequate time to discuss HCC surveillance (37 of 139 [26.6%] vs 2 of 48 [4.2%]; *P* = .001), concerns about patients’ transportation barriers to complete surveillance testing (13 of 139 [9.4%] vs 0 of 48 [0%]; *P* = .03), and concerns of patients’ potential out-of-pocket costs (25 of 139 [18.0%] vs 2 of 48 [4.2%]; *P* = .02).

**Table 3.** Perceived Barriers to Hepatocellular Carcinoma Surveillance by Clinician Specialty

Perceived barriers to ordering HCC surveillance	Respondents, No. (%)^a^		*P* value
	Internal medicine or family medicine	Gastroenterology and hepatology	
**Not enough time or have more important things to manage in clinic than to discuss liver cancer screening with patients (n = 187)**			
No barriers	102 (73.4)	46 (95.8)	.001
Barriers reported	37 (26.6)	2 (4.2)	
**Difficulty discussing liver cancer screening with patients due to language barriers (n = 187)**			
No barriers	131 (92.9)	42 (91.3)	.72
Barriers reported	10 (7.1)	4 (8.7)	
**Shortage of radiology facilities in my area to perform liver cancer screening tests (n = 188)**			
No barriers	129 (91.5)	43 (91.5)	>.99
Barriers reported	12 (8.5)	4 (8.5)	
**Patients often do not complete liver cancer screening tests that have been ordered (n = 185)**			
No barriers	126 (91.3)	45 (95.7)	.32
Barriers reported	12 (8.7)	2 (4.3)	
**Patients often lack transportation to be able complete liver cancer screening testing that is ordered (n = 187)**			
No barriers	126 (90.6)	48 (100.0)	.03
Barriers reported	13 (9.4)	0	
**Concerns about patients’ out-of-pocket costs (n = 187)**			
No barriers	114 (82.0)	46 (95.8)	.02
Barriers reported	25 (18.0)	2 (4.2)	
**Difficulty arranging follow-up diagnostic testing for patients who have a positive screening test (n = 186)**			
No barriers	126 (90.0)	43 (93.5)	.48
Barriers reported	14 (10.0)	3 (6.5)	
**Difficulty with arranging treatment for patients diagnosed with liver cancer (n = 187)**			
No barriers	131 (94.2)	46 (95.8)	.67
Barriers reported	8 (5.8)	2 (4.2)	

^a^ Not all respondents answered all questions. Calculation of proportions were based on the total number of clinicians who answered each question. The number in parentheses in the first column indicates the total number of respondents for each category.

We also assessed clinician beliefs and perceptions about HCC surveillance and how that may have contributed to barriers in effective HCC surveillance (Table 4). PCCs, compared with gastroenterology and hepatology clinicians, were more likely to report difficulty identifying which patients have cirrhosis (82 of 141 [58.2%] vs 5 of 48 [10.4%]; *P* < .001) and more likely to feel that they were not up-to-date with current guidelines for HCC surveillance (87 of 139 [62.6%] vs 5 of 48 [10.4%]; *P* < .001). Language barriers, shortage of radiology facilities, concerns that patients often do not complete surveillance tests that are ordered, difficulty arranging follow-up diagnostic testing for those with a positive HCC screening test, and difficulty arranging treatment for patient diagnosed with HCC were reported in 10% or less of clinicians and did not differ significantly between PCCs and gastroenterology and hepatology clinicians (Table 4).

**Table 4.** Beliefs, Attitudes, and Perceptions Toward Hepatocellular Carcinoma Surveillance by Clinician Specialty

Beliefs, attitudes, and perceptions	Respondents, No. (%)^a^		*P* value
	Internal medicine or family medicine	Gastroenterology and hepatology	
**I want to order liver cancer screening but have difficulty knowing which patients with liver disease have cirrhosis (n = 188)**			
Strongly agree or agree	82 (58.2)	5 (10.4)	<.001
Strongly disagree or disagree	59 (41.8)	43 (89.6)	
**I do not order liver cancer screening because current screening tools are suboptimal and miss many cancers (n = 187)**			
Strongly agree or agree	11 (7.9)	0	.048
Strongly disagree or disagree	129 (92.1)	47 (100.0)	
**I do not order liver cancer screening because there are not any effective treatments available (n = 187)**			
Strongly agree or agree	1 (0.7)	0	.56
Strongly disagree or disagree	138 (99.3)	48 (100.0)	
**I do not order liver cancer screening because it does not change survival (n = 188)**			
Strongly agree or agree	1 (0.7)	1 (2.1)	.43
Strongly disagree or disagree	139 (99.3)	47 (97.9)	
**I do not order liver cancer screening because it is the responsibility of other clinicians to order and follow up on liver cancer screening (n = 188)**			
Strongly agree or agree	27 (19.3)	7 (14.6)	.47
Strongly disagree or disagree	113 (80.7)	41 (85.4)	
**I do not feel that I am up to date with current guidelines for liver cancer screening (n = 187)**			
Strongly agree/agree	87 (62.6)	5 (10.4)	<.001
Strongly disagree or disagree	52 (37.4)	43 (89.6)	
**Liver cancer screening is effective at detecting tumors at an early stage (n = 187)**			
Strongly agree or agree	126 (90.6)	45 (93.8)	.51
Strongly disagree or disagree	13 (9.4)	3 (6.3)	
**Liver cancer screening is cost-effective in patients with cirrhosis (n = 187)**			
Strongly agree or agree	127 (91.4)	44 (91.7)	.95
Strongly disagree or disagree	12 (8.6)	4 (8.3)	
**Not performing liver cancer screening poses malpractice liability (n = 185)**			
Strongly agree or agree	111 (81.0)	47 (97.9)	.004
Strongly disagree or disagree	26 (19.0)	1 (2.1)	
**Better data are needed to evaluate benefits of liver cancer screening in patients with cirrhosis (n = 182)**			
Strongly agree or agree	74 (54.4)	16 (34.8)	.02
Strongly disagree or disagree	62 (45.6)	30 (65.2)	
**Better data are needed to evaluate harms of liver cancer screening in patients with cirrhosis (n = 181)**			
Strongly agree or agree	65 (48.5)	16 (34.0)	.09
Strongly disagree or disagree	69 (51.5)	31 (66.0)	
**Better education for primary care clinicians about liver cancer and liver cancer screening is needed (n = 188)**			
Strongly agree or agree	134 (95.7)	48 (100.0)	.15
Strongly disagree or disagree	6 (4.3)	0	

^a^ Not all respondents answered all questions. Calculation of proportions were based on the total number of clinicians who answered each question. The number in parentheses in the first column indicates the total number of respondents for each category.

### Clinician Attitudes Toward Surveillance

Clinician attitudes toward HCC surveillance, stratified by clinician subspecialty, are reported in Table 4. A higher proportion of PCCs, compared with gastroenerology and hepatology clinicians, believe that current screening tools are suboptimal and miss many HCC (11 of 140 [7.9%] vs 0 of 47 [0%]; *P* = .048) as well as endorsing the need for better data to evaluate the benefits of HCC surveillance in patients with cirrhosis (74 of 136 [54.4%] vs 16 of 46 [34.8%]; *P* = .02). Gastroenterology and hepatology clinicians were more likely than PCCs to agree that lack of HCC surveillance poses medical malpractice liability (47 of 48 [97.9%] vs 111 of 137 [81.0%]; *P* = .004). Nearly all clinicians, regardless of specialty, did not believe the lack of effective HCC treatments or lack of association with survival contributed to HCC surveillance underuse. Similarly, nearly all clinicians agreed that HCC surveillance is effective at detecting tumors at an early stage and that HCC surveillance is cost-effective in patients with cirrhosis. Finally, nearly all clinicians agreed that better education for PCCs about HCC surveillance is needed.

### Impact of COVID-19 on HCC Surveillance

Over 80% of clinicians across both primary care and gastroenterology and hepatology agreed that HCC surveillance was delayed during the pandemic (Table 5). Over one-third of gastroenterology and hepatology clinicians and PCCs believed the transition to telehealth for patients with cirrhosis was difficult. Compared with gastroenterology and hepatology clinicians, PCCs were less likely to have an effective mechanism to track patients with cirrhosis who missed HCC surveillance appointments during the COVID-19 pandemic (12 of 135 [8.9%] vs 10 of 45 [22.2%]; *P* = .02). Currently, only 62 of 136 PCCs (45.6%) and 27 of 45 gastroenterology and hepatology clinicians (60.0%) reported that all their patients who need HCC surveillance can get their testing scheduled in a timely manner. Furthermore, a large proportion of clinicians felt that COVID-19 pandemic–related delays and barriers associated with HCC surveillance continue to persist at this time, more so among PCCs vs gastroenterology and hepatology clinicians (67 of 135 [49.6%] vs 14 of 45 [31.1%]; *P* = .03) (Table 5).

**Table 5.** COVID-19 Pandemic–Related Disruptions Associated With Hepatocellular Carcinoma Surveillance

COVID-19 pandemic–related disruptions	Respondents, No. (%)^a^		*P* value
	Internal medicine or family medicine	Gastroenterology and hepatology	
**During the pandemic, my patients with cirrhosis often missed their regularly scheduled appointments (n = 182)**			
Strongly agree or agree	116 (84.7)	41 (91.1)	.28
Strongly disagree or disagree	21 (15.3)	4 (8.9)	
**Patients with cirrhosis in my clinic were able to transition to telehealth visits without difficulty (n = 178)**			
Strongly agree or agree	76 (57.1)	29 (64.4)	.39
Strongly disagree or disagree	57 (42.9)	16 (35.6)	
**Liver cancer screening was delayed or postponed during the pandemic due to limitations of in-person visits (n = 181)**			
Strongly agree or agree	113 (83.7)	38 (82.6)	.86
Strongly disagree or disagree	22 (16.3)	8 (17.4)	
**I have an effective mechanism to keep track of patients with cirrhosis who have missed their liver cancer screening during the pandemic to make sure they are rescheduled (n = 180)**			
Strongly agree or agree	12 (8.9)	10 (22.2)	.02
Strongly disagree or disagree	123 (91.1)	35 (77.8)	
**Currently, all my patients who need liver cancer screening can get their testing scheduled without delays (n = 181)**			
Strongly agree or agree	62 (45.6)	27 (60.0)	.09
Strongly disagree or disagree	74 (54.4)	18 (40.0)	
**Currently, all the pandemic related delays or barriers in scheduling liver cancer screening have been completely resolved in my practice setting (n = 180)**			
Strongly agree or agree	68 (50.4)	31 (68.9)	.03
Strongly disagree or disagree	67 (49.6)	14 (31.1)	

^a^ Not all respondents answered all questions. Calculation of proportions were based on the total number of clinicians who answered each question. The number in parentheses in the first column indicates the total number of respondents for each category.

## Discussion

Among PCCs and gastroenterology and hepatology clinicians across 5 safety-net health systems in 5 distinct geographic regions in the US, we identified important potentially modifiable barriers contributing to low HCC surveillance in patients with cirrhosis. First, respondents, particularly PCCs, had suboptimal knowledge regarding the appropriate timing of HCC surveillance as well as appropriate choice of surveillance modality. Specifically, when compared with gastroenterology and hepatology clinicians, PCCs were more likely to cite challenges with accurately identifying patients with cirrhosis as well as feeling not up-to-date with current HCC surveillance guidelines. These observations align with prior studies citing gaps in knowledge contributing to underuse of HCC surveillance, but is unique in focusing on safety-net clinicians and inclusive of both PCCs and gastroenterologists and hepatologists.^13,15,29^ For example, among 131 PCCs surveyed at a single center health system, Dalton-Fitzgerald^13^ noted that 68% of respondents reported feeling that they were not up-to-date with HCC surveillance guidelines. Similarly, among 391 North Carolina PCCs surveyed, only 45% reported ordering HCC surveillance in their patients with cirrhosis, and among those not ordering surveillance, 24% reported being unaware of HCC surveillance recommendations in patients with cirrhosis.^29^ It is also interesting that survey respondents with less than 10 years of experience had the highest HCC knowledge score, and this could reflect individuals that have more recently completed training and therefore may be more up to date with HCC surveillance guidelines. While drivers of suboptimal HCC surveillance are multifactorial, incorporating educational interventions or user-friendly decision support tools^30^ that can improve HCC surveillance knowledge and disseminate updated HCC guidelines, particularly among PCCs, will be a necessary component of any program to improve HCC surveillance. In fact, nearly 96% of PCCs who responded to our survey agreed that better education about HCC and HCC surveillance are needed for PCCs. While we acknowledge the multifactorial challenges that lead to suboptimal HCC surveillance, effective delivery of education to improve cirrhosis and HCC surveillance knowledge that specifically targets specific gaps or misperceptions is a low-hanging fruit that could improve HCC surveillance use among safety-net health systems. Furthermore, delivery of education to clinicians who use multiple modalities and offered on a recurring basis may be more effective than a 1-time intervention. However, we acknowledge the challenge of implementing this type of intervention in PCC settings, especially on a recurring basis, given that PCCs have multiple conditions and practice guidelines to stay up to date with across the spectrum of medical care for adults.

In addition to knowledge gaps, 41.6% of respondents in our study reported at least 1 perceived barrier to ordering HCC surveillance, with inadequate time to discuss HCC surveillance during clinical encounters, concerns for patients’ out-of-pocket costs, and challenges in arranging follow-up diagnostic testing being the top 3 barriers reported by PCCs. These reported challenges highlight the complex health system level factors that are not unique to PCCs nor unique to safety-net health systems affecting HCC surveillance in patients with cirrhosis.^10^ In fact, the uniqueness of our study focusing on safety-net populations emphasizes the complex operational challenges that exist in underresourced settings. Programs aiming to improve HCC surveillance particularly in safety-net health systems must also address the health system factors in parallel with clinician and patient specific challenges that exist. We additionally assessed differences in beliefs, attitudes, and perceptions toward HCC surveillance between PCCs and gastroenterology and hepatology clinicians. Nearly 8% of PCCs believed that current HCC surveillance tools are suboptimal and 55% believe that better data are needed to demonstrate the benefits of HCC surveillance in patients with cirrhosis, both significantly greater than gastroenterology and hepatology clinicians. These observations further emphasize the need for more effective means to share knowledge and disseminate relevant literature and guidelines with PCCs in particular about the outcomes and benefits of HCC surveillance among patients with cirrhosis.^6,31,32^

While important clinician-level factors potentially contributing to underuse of HCC surveillance were identified, a unique aspect of our study is the evaluation of COVID-19 pandemic–related disruptions in further exacerbating barriers to timely HCC surveillance among safety-net health systems. Most clinicians reported that patients with cirrhosis often missed appointments and HCC surveillance was delayed due to limitations of in-person visits. These observations confirm existing reports and concerns about the long-term outcomes of delays in cirrhosis care following onset of the COVID-19 pandemic.^33,34,35^ However, even more concerning, among survey respondents in the current study, it is clear that despite being over 3 years post onset of the pandemic, the disruptions in cirrhosis care and HCC surveillance in particular remain, and most respondents do not have an effective mechanism to track and re-engage patients who missed HCC surveillance during the pandemic. This is particularly concerning given that populations who use safety-net health systems have already experienced barriers in timely access to health care even before the onset of the COVID-19 pandemic. These barriers stress the importance of considering interventions to improve HCC surveillance, including electronic medical record dashboards and reminder systems, radiology recall systems, or organized health-system level outreach strategies.^36,37,38,39^

### Strengths and Limitations

A strength of our study, which focused specifically on safety-net health systems, was that it incorporated survey responses among both PCCs and gastroenterology and hepatology specialists across diverse geographical regions. In addition to assessing knowledge, attitudes, perceived barriers, and beliefs that may affect use of HCC surveillance, the current study is unique in also assessing the COVID-19 pandemic–related impact on HCC surveillance and how those disruptions have persisted into the present day.

However, certain limitations should be acknowledged. Given the survey-based approach, the possibility of recall bias should be considered when interpreting the data. Overall, with the 25.5% response rate, nonresponse bias should be considered as well as social desirability bias, such that respondents answer questions in a socially desirable manner rather than reflecting true practice patterns or beliefs. In addition, some respondents only answered some questions or may have been hesitant to report challenges that were felt to be unique to their health system. We attempted to address some of these concerns by ensuring survey respondents remained anonymous. However, nonresponse and missing data for certain questions may reflect social desirability bias. Another potential limitation is that clinicians who responded to the survey may have been newly employed or may have recently moved from different institutions and thus their responses regarding challenges and barriers, especially as they are associated with health system factors, may be influenced by their experiences at prior places of patient care. Finally, our study collected perspectives of clinicians but does not reflect other challenges associated with surveillance implementation, including patient-reported barriers.^40^

## Conclusions

In this survey study, we identified important gaps in knowledge and perceived challenges and barriers to effective use of HCC surveillance among patients with cirrhosis. Despite these concerning observations, these data clearly demonstrated potentially modifiable factors, and increased efforts toward targeted dissemination of HCC surveillance education, particularly among PCCs, is a critical component of efforts to improve HCC surveillance among patients with cirrhosis. We also observed that pandemic-related disruptions in clinical care affecting HCC surveillance have persisted despite being more than 3 years post onset of the COVID-19 pandemic. These findings highlighted that in addition to targeted education, parallel health system–level interventions must be pursued to address the barriers to the availability of resources and infrastructure needed to ensure close monitoring of patients with cirrhosis.

## Notes

Supplement 1. **eMethods.** Liver Cancer Screening Clinician Survey **eTable 1.** Characteristics of the Cohort **eTable 2.** HCC Knowledge Assessment

Supplement 2.
Data Sharing Statement
