# Supplementary material for: Clinician-Level Knowledge and Barriers to Hepatocellular Carcinoma Surveillance
Source: JAMA Netw Open. 2024 May 14;7(5):e2411076. doi: 10.1001/jamanetworkopen.2024.11076 (PMC11094557; doi:10.1001/jamanetworkopen.2024.11076)
Supplement: Supplement 1. — eMethods. Liver Cancer Screening Clinician Survey eTable 1. Characteristics of the Cohort eTable 2. HCC Knowledge Assessment [file jamanetwopen-e2411076-s001.pdf]

Supplemental Online Content

Wong RJ, Jones PD, Niu B, et al. Clinician-level knowledge and barriers to hepatocellular carcinoma surveillance in the US. *JAMA Netw Open*. 2024;7(5):e2411076. doi:10.1001/jamanetworkopen.2024.11076

eMethods. Liver Cancer Screening Clinician Survey
eTable. Characteristics of the Cohort
eTable 2. HCC Knowledge Assessment

This supplemental material has been provided by the authors to give readers additional information about their work.

© 2024 Wong RJ et al. *JAMA Network Open.*

Liver Cancer Screening Clinician Survey

Practice Characteristics

1. How many years have you been practicing medicine after completing your most recent training?
    a. Still in training (e.g. residency, fellowship)
    b. < 5 years
    c. 5 – 10 years
    d. 10 – 20 years
    e. > 20 years

2. Approximately how many total patients do you see in a typical week?
    a. < 25 patients
    b. 25 – 49 patients
    c. 50 – 74 patients
    d. 75 - 99 patients
    e. 100 or more patients

3. Approximately how many cirrhosis patients do you see in a typical week:
    a. < 5 cirrhosis patients
    b. 5 – 10 cirrhosis patients
    c. 10 – 20 cirrhosis patients
    d. > 20 cirrhosis patients

4. Approximately what proportion of your cirrhosis patients have had an ultrasound performed for liver cancer screening in the past 6 months? ____________%

5. Approximately what proportion of your cirrhosis patients have had an alpha fetoprotein (AFP) test completed for liver cancer screening in the past 6 months? ____________%

Liver Cancer Screening and Surveillance Knowledge

1. You see a 50-year-old man with newly diagnosed cirrhosis in your clinic. When would you recommend starting HCC screening?
    a. Start screening now
    b. Start screening at next visit in 6 months
    c. Start screening at next visit in 12 months
    d. HCC screening is not needed at this time

2. You are continuing to follow a patient with nonalcoholic steatohepatitis (NASH) related cirrhosis in your clinic who has just completed an ultrasound for HCC screening. The

© 2024 Wong RJ et al. *JAMA Network Open.*

ultrasound does not show any liver masses. When would you recommend the next ultrasound be performed?

a. No further ultrasounds are needed
b. Ultrasound in 3 months
c. Ultrasound in 6 months
d. Ultrasound in 12 months
e. CT or MRI in 3 months
f. CT or MRI in 6 months
g. CT or MRI in 12 months

3. For each of the following patient scenarios, indicate whether you would recommend HCC screening, and if so, what screening modality you would most likely recommend. Please assume compensated cirrhosis when cirrhosis is mentioned, and assume no other co-morbidities are present.

I. 65-year-old woman with non-alcoholic fatty liver disease (NAFLD) <u>without</u> evidence of cirrhosis
   a. No HCC screening recommended
   b. Alpha fetoprotein only
   c. Ultrasound only
   d. Ultrasound and alpha fetoprotein
   e. CT and/or MRI

II. 50-year-old man with hepatitis C virus (HCV) related cirrhosis
   a. No HCC screening recommended
   b. Alpha fetoprotein only
   c. Ultrasound only
   d. Ultrasound and alpha fetoprotein
   e. CT and/or MRI

III. 70-year-old man with HCV and alcohol-related liver disease <u>without</u> cirrhosis
   a. No HCC screening recommended
   b. Alpha fetoprotein only
   c. Ultrasound only
   d. Ultrasound and alpha fetoprotein
   e. CT and/or MRI

IV. 55-year-old man with NASH related cirrhosis
   a. No HCC screening recommended
   b. Alpha fetoprotein only
   c. Ultrasound only
   d. Ultrasound and alpha fetoprotein
   e. CT and/or MRI

Liver Cancer Attitudes and Perception

© 2024 Wong RJ et al. *JAMA Network Open.*

Please indicate whether you agree or disagree with the following statements about liver cancer screening.

1. I want to order liver cancer screening but have difficulty knowing which patients with liver disease have cirrhosis.
   a. Strongly agree
   b. Agree
   c. Disagree
   d. Strongly disagree
2. I do not have enough time or have more important things to manage in clinic than to discuss liver cancer screening with patients.
   a. Strongly agree
   b. Agree
   c. Disagree
   d. Strongly disagree
3. I often have difficulty discussing liver cancer screening with patients due to language barriers
   a. Strongly agree
   b. Agree
   c. Disagree
   d. Strongly disagree
4. There is a shortage of radiology facilities in my area to perform liver cancer screening tests.
   a. Strongly agree
   b. Agree
   c. Disagree
   d. Strongly disagree
5. I do not order liver cancer screening testing because patients often do not complete the examination after it has been ordered
   a. Strongly agree
   b. Agree
   c. Disagree
   d. Strongly disagree
6. I do not order liver cancer screening testing because patients often lack transportation to be able complete the examination after it has been ordered.
   a. Strongly agree
   b. Agree
   c. Disagree
   d. Strongly disagree
7. I do not order liver cancer screening testing given patients' concerns for out-of-pocket costs.
   a. Strongly agree
   b. Agree
   c. Disagree

© 2024 Wong RJ et al. *JAMA Network Open.*

    d. Strongly disagree
8. I do not order liver cancer screening because current screening tools are suboptimal and miss many cancers.
    a. Strongly agree
    b. Agree
    c. Disagree
    d. Strongly disagree
9. I do not order liver cancer screening because I have a difficult time arranging follow-up diagnostic testing for patients who have a positive screening test.
    a. Strongly agree
    b. Agree
    c. Disagree
    d. Strongly disagree
10. I do not order liver cancer screening because I have a difficult time arranging treatment for patients diagnosed with liver cancer.
    a. Strongly agree
    b. Agree
    c. Disagree
    d. Strongly disagree
11. I do not order liver cancer screening because there are not any effective treatments available.
    a. Strongly agree
    b. Agree
    c. Disagree
    d. Strongly disagree
12. I do not order liver cancer screening because it doesn't change survival.
    a. Strongly agree
    b. Agree
    c. Disagree
    d. Strongly disagree
13. I do not order liver cancer screening because it is the responsibility of other providers to order and follow up on liver cancer screening.
    a. Strongly agree
    b. Agree
    c. Disagree
    d. Strongly disagree
14. I do not feel that I am up to date with current guidelines for liver cancer screening.
    a. Strongly agree
    b. Agree
    c. Disagree
    d. Strongly disagree

© 2024 Wong RJ et al. *JAMA Network Open.*

<u>Liver Cancer Screening Beliefs</u>

How effective do you believe the following modalities are for finding liver cancer at an early stage in patients with cirrhosis?

1. Clinical physical examination
   a. Very effective
   b. Somewhat effective
   c. Not effective
2. Liver enzymes testing
   a. Very effective
   b. Somewhat effective
   c. Not effective
3. Alpha fetoprotein alone
   a. Very effective
   b. Somewhat effective
   c. Not effective
4. Ultrasound alone
   a. Very effective
   b. Somewhat effective
   c. Not effective
5. Ultrasound combined with alpha fetoprotein
   a. Very effective
   b. Somewhat effective
   c. Not effective
6. Single-phase CT scan (one often completed in emergency department)
   a. Very effective
   b. Somewhat effective
   c. Not effective
7. Contrast enhanced multi-phase CT scan
   a. Very effective
   b. Somewhat effective
   c. Not effective
8. Contrast enhanced dynamic multi-phase MRI scan
   a. Very effective
   b. Somewhat effective
   c. Not effective

Please indicate whether you agree or disagree with the following statements about liver cancer screening:

1. Liver cancer screening is effective at detecting tumors at an early stage.
   a. Strongly agree
   b. Agree
   c. Disagree

© 2024 Wong RJ et al. *JAMA Network Open.*

d. Strongly disagree
2. Liver cancer screening is cost-effective in patients with cirrhosis.
    a. Strongly agree
    b. Agree
    c. Disagree
    d. Strongly disagree
3. Not performing liver cancer screening poses malpractice liability.
    a. Strongly agree
    b. Agree
    c. Disagree
    d. Strongly disagree
4. Better data are needed to evaluate benefits of liver cancer screening in patients with cirrhosis.
    a. Strongly agree
    b. Agree
    c. Disagree
    d. Strongly disagree
5. Better data are needed to evaluate harms of liver cancer screening in patients with cirrhosis.
    a. Strongly agree
    b. Agree
    c. Disagree
    d. Strongly disagree
6. Better education for primary care providers about liver cancer and liver cancer screening is needed.
    a. Strongly agree
    b. Agree
    c. Disagree
    d. Strongly disagree

COVID-19 Pandemic Related Disruptions in Liver Cancer Screening

Please indicate whether you agree or disagree with the following statements about the impact of COVID-19 pandemic on your practice:

1. During the pandemic, my cirrhosis patients often missed their regularly scheduled appointments.
    a. Strongly agree
    b. Agree
    c. Disagree
    d. Strongly disagree
2. Cirrhosis patients in my clinic were able to transition to telehealth visits without difficulty

© 2024 Wong RJ et al. *JAMA Network Open.*

    a. Strongly agree
    b. Agree
    c. Disagree
    d. Strongly disagree
3. Liver cancer screening was delayed or postponed during the pandemic due to limitations of in-person visits.
    a. Strongly agree
    b. Agree
    c. Disagree
    d. Strongly disagree
4. I have an effective mechanism to keep track of cirrhosis patients who have missed their liver cancer screening during the pandemic to make sure they are rescheduled.
    a. Strongly agree
    b. Agree
    c. Disagree
    d. Strongly disagree
5. Currently, all my patients who need liver cancer screening can get their testing scheduled without delays.
    a. Strongly agree
    b. Agree
    c. Disagree
    d. Strongly disagree
6. Currently, all the pandemic related delays or barriers in scheduling liver cancer screening have been completely resolved in my practice setting.
    a. Strongly agree
    b. Agree
    c. Disagree
    d. Strongly disagree

<u>Provider Characteristics</u>

1. What is your gender?
    a. Male
    b. Female
    c. Other
2. Which best describes your race?
    a. American Indian or Alaska Native
    b. Asian
    c. Black or African American
    d. Native Hawaiian or other Pacific Islander
    e. White
    f. Other

© 2024 Wong RJ et al. *JAMA Network Open.*

3. Do you consider yourself to be Hispanic or Latino?
   a. Yes
   b. No
4. What is your age?
   a. Specify number: _______________
5. Which best describes your highest level of training/certification?
   a. MD/DO, still in training (e.g. resident or fellow)
   b. MD/DO, completed training, but not board certified
   c. MD/DO, board certified in internal medicine or family medicine
   d. MD/DO, board certified in gastroenterology and/or transplant hepatology
   e. NP/PA, internal medicine or family medicine
   f. NP/PA, gastroenterology and/or hepatology
   g. Other: Please specify:_________________
6. Which best describes the primary practice/specialty you spend the majority of your clinical time?
   a. Currently in training (e.g. resident or fellow)
   b. Internal medicine or family medicine
   c. Gastroenterology or hepatology
   d. Other: Please specify:________________

© 2024 Wong RJ et al. *JAMA Network Open.*

eTable 1. Characteristics of the Cohort

	Respondents, No. (%)
Provider Specialty (n=237)	
Internal Medicine/Family Medicine	142 (59.9)
Gastroenterology/Hepatology	48 (20.3)
Currently in Training	39 (16.5)
Other	8 (3.4)
Gender (n=233)	
Men	85 (36.5)
Women	148 (63.5)
Race (n=232)	
African American	18 (7.8)
Asian	50 (21.6)
Other	17 (7.3)
White	147 (63.4)
Ethnicity (n=234)	
Hispanic	12 (5.1)
Non-Hispanic	222 (94.9)
Age (mean and standard deviation)	43.7 (11.5)
Provider Type/Training (n=236)	
MD/DO	190 (80.5)
NP/PA	46 (19.5)
Provider Experience (n=310)	
< 10 years experience	161 (51.9)
10-20 years experience	67 (21.6)
> 20 years experience	82 (26.5)
Number of patients seen in a typical week (n=309)	
< 25 patients	58 (18.8)
25 - 49 patients	97 (31.4)
50 or more patients	154 (49.8)

Note: The number in parentheses for each category indicates the total number of respondents

eTable 2. HCC Knowledge Assessment

HCC Knowledge was assessed based on correctly identifying when to implement HCC surveillance and the correct diagnostic modality in the following 6 clinical scenarios:

1. A 50-y-old man with newly diagnosed cirrhosis
2. A patient with nonalcoholic steatohepatitis (NASH) related cirrhosis who has just completed an ultrasound for HCC screening that does not show any liver masses.
3. A 65-y-old woman with non-alcoholic fatty liver disease (NAFLD) without evidence of cirrhosis
4. A 50-y-old man with hepatitis C virus (HCV) related cirrhosis
5. A 70-y-old man with HCV and alcohol-related liver disease without cirrhosis
6. A 55-y-old man with NASH related cirrhosis

© 2024 Wong RJ et al. *JAMA Network Open.*
